# Cumulative comorbidity between neurodevelopmental, internalising, and externalising disorders in childhood: a network approach

**DOI:** 10.1007/s00787-023-02312-7

**Published:** 2023-10-10

**Authors:** Oliver J. Watkeys, Kirstie O’Hare, Kimberlie Dean, Kristin R. Laurens, Felicity Harris, Vaughan J. Carr, Melissa J. Green

**Affiliations:** 1 School of Clinical Medicine, Discipline of Psychiatry and Mental Health, Faculty of Medicine and Health, University of New South Wales (UNSW), Level 1, AGSM Building, Kensington Campus, Sydney, Australia; 2 Neuroscience Research Australia, Sydney, Australia; 3 Justice Health and Forensic Mental Network, Matraville, Australia; 4 School of Psychology and Counselling, Queensland University of Technology (QUT), Brisbane, Australia; 5 Department of Psychiatry, Monash University, Melbourne, Australia

**Keywords:** Cumulative comorbidity, Community detection, Sex differences, Children, Network analysis

## Abstract

Cumulative comorbidity of mental disorders is common, but the extent and patterns of comorbid psychopathology in childhood are not well established. The current study aimed to elucidate the emergent patterns of cumulative mental disorder comorbidity in children using network analysis of diagnoses recorded between birth and age 12 years. Participants were 90,269 children (mean age 12.7 years; 51.8% male) within the New South Wales Child Development Study (NSW-CDS)—a longitudinal record-linkage cohort study of Australian children born in NSW between 2002 and 2005. Binary indicators for eight types of mental disorder were derived from administrative health records. Patterns of conditional association between mental disorders were assessed utilising network analysis. Of 90,269 children, 2268 (2.5%) had at least one mental disorder by age 12 years; of the 2268 children who had at least one mental disorder by age 12 years, 461 (20.3%) were diagnosed with two or more different disorders out of the eight disorder types included in analyses. All disorders were either directly or indirectly interconnected, with childhood affective and emotional disorders and developmental disorders being most central to the network overall. Mental disorder nodes aggregated weakly (modularity = 0.185) into two communities, representative of internalising and externalising disorders, and neurodevelopmental and sleep disorders. Considerable sex differences in the structure of the mental disorder comorbidity networks were also observed. Developmental and childhood affective and emotional disorders appear to be key to mental disorder comorbidity in childhood, potentially reflecting that these disorders share symptoms in common with many other disorders.

**Supplementary Information:**

The online version contains supplementary material available at 10.1007/s00787-023-02312-7.

## Introduction

Cumulative mental disorder comorbidity, in which an individual experiences multiple mental disorders over their lifetime, is pervasive in the general population [36] and has been linked to poor quality of life and reduced global functioning [21, 40]. Mental disorder comorbidity is also pervasive in childhood, such that one-third of affected children will be diagnosed with more than one type of mental disorder [30, 47]. The pervasiveness of mental disorder comorbidity has challenged current mental disorder taxonomies and may reflect indistinct boundaries between psychiatric diagnoses, clinical heterogeneity within disorders (increasing the rate of misdiagnosis), and arbitrary distinctions between normality and pathology [9, 45]. Contemporary proposals of a single dimension of psychopathology (the *p-*factor) [9, 29] can account for the typical set of subdimensions (e.g. internalising, externalising, and thought disorder) emerging in population-level research of mental disorders whilst maintaining the essentialist notion of an underlying pathology which causes psychological disorders [9, 10, 26]. This model has arisen alongside the (not incompatible) Hierarchical Taxonomy of Psychopathology (HiTOP) [29], a multidimensional model that provides an alternative framework for the quantitative classification of mental illness.

An alternative theoretical perspective conceptualises psychopathology as a network of causally interconnected symptoms [3, 4, 26]. This theory proposes that external causes of psychopathology can trigger an initial symptom, which can then lead to further (potentially mutually reinforcing) symptoms, meaning that mental illness can persist after the removal of the proximal cause [3]. According to this theory, comorbidity is not brought about by the co-occurrence of distinct disorders, or underlying transdiagnostic liability (i.e. *p*-factor), but by “bridge” symptoms shared by multiple disorders [3, 4]. Network approaches to the study of psychopathology identify the most influential symptoms (or nodes) which bridge the associations between groups of symptoms, and are thereby the most influential to the network in terms of “bridge centrality”. For example, a number of recent studies have found physical symptoms (e.g. restlessness, appetite changes, and dizziness) to be the strongest bridge symptoms between anxiety, depression, and eating disorders [25, 31]. Network approaches are flexible as they can model (potentially mutually) reinforcing associations between individual symptoms of disorders or disorders themselves as nodes within a network, thus providing a means of examining relationships between ostensibly distinct psychopathologies and a systematic overview of mental disorder comorbidity [4]. For example, a recent network analysis of mental illness in children revealed pervasive conditional associations between eight different mental disorders which clustered into internalising and externalising disorders [35]. Other network analyses of comorbidities among children with autism spectrum disorders reported conditional associations of internalising disorders, hyperkinetic disorders, and intellectual disability [18, 32]. However, to our knowledge, there have not been any network analyses examining comorbidity between internalising, externalising, neurodevelopmental, intellectual and sleep disorders among children in the general population.

The current study aimed to elucidate population-level emergent patterns of cumulative mental disorder comorbidity, including neurodevelopmental disorders, in a cohort of children using network analysis. We specifically examined cumulative comorbidity between groups of mental disorders (e.g. by including conduct disorders, anxiety/phobic disorders, neurodevelopmental disorders as nodes) using traditional network analysis and centrality measures, before evaluating how these broad groups of disorders aggregate into larger communities of disorders, and determining which mental disorder categories might “bridge” these disorder communities. Mental disorder categories were selected on the basis of symptom similarity and levels of comorbidity [19, 34, 37] as recognised in International Classification of Diseases 10th Edition Australian Modification (ICD-10-AM) diagnostic nomenclature. This approach facilitated investigation of mental disorder comorbidity across the spectrum of psychopathology in childhood, incorporating multiple disorders under broader diagnostic groups enabling examination of disorders which would otherwise be too rare to reliably investigate in isolation. It was hypothesised that all mental and neurodevelopmental disorders would be associated with one another in childhood, and that network models may be different for boys and girls. Furthermore, we anticipated that mental disorders in children would cluster into internalising, externalising, and neurodevelopmental groups, consistent with the stronger patterns of mental disorder comorbidity within these disorder subgroups that have been reported previously [10, 35].

## Methods

### Study cohort

A total of 90,269 children (mean age on 31st December 2016 = 12.7 years; standard deviation [SD] = 0.4) were drawn from the New South Wales Child Development Study (NSW-CDS: http://nsw-cds.com.au/), an intergenerational, longitudinal record-linkage study comprising a population of 91,635 children born between 2002 and 2004 [8, 20]. Children were excluded if their birth record indicated that they had an older sibling in the cohort, in order to remove any non-independent observations from our study (*n* = 1366). Record linkages were conducted by the NSW Centre for Health Record Linkage (http://www.cherel.org.au/) with source records available from 1994 to 2016, inclusive. Record-linkage accuracy was high, such that the false positive rate was only 0.5% [20]. Ethical approval was provided by the NSW Population and Health Services Research Ethics Committee (HREC/15/CIPHS/21).

### Mental disorder indicators

Indices of mental disorders for children were derived from health records, including the NSW Ministry of Health’s Admitted Patient Data Collection (APDC; 2001–2016), Mental Health Ambulatory Data Collection (MHAMB; 2001–2015), and Emergency Department Data Collection (EDDC; 2005–2016). Each of these databases captures information from distinct branches of NSW Health’s service provision. The APDC and EDDC reflect inpatient admissions and emergency presentations to hospitals, respectively. The MHAMB captures outpatient community-based treatment, crisis assessment, as well as hospital-based outreach and in-reach services [1]. Binary indicators for eight different mental disorder categories commonly diagnosed in childhood (anxiety and phobic disorders, child affective and emotional disorders, stress reactions, hyperkinetic disorders, conduct disorders, neurodevelopmental disorders, intellectual disability, and sleep disorders) were based on recorded ICD-10-AM codes. The full list of ICD-10-AM codes comprised within each of the mental disorder categories and their frequencies in the child cohort are presented in Table S1. Data were restricted to diagnoses that occurred prior to 12 years of age, as not all children in the cohort had reached 12 years of age by the end of the observation period.

### Analysis

All analyses were completed in RStudio (2022.02.3) using R (v4.0.3). The childhood mental disorder cumulative comorbidity network was estimated using the *bootnet* [16] and *IsingFit* packages [43] which implement an l1-regularised form of logistic regression designed for the network analysis with binary variables. The resulting matrix of undirected edge-weights was plotted using the *qgraph* package [17]. The degree of interconnectedness between each mental disorder node was assessed using ‘Strength’ centrality—a score reflecting the number and strength of edges (conditional associations) for each node in the network. Robustness of edge-weights and centrality metrices were estimated via non-parametric bootstrapping and case-dropping, respectively, using the *bootnet* package, treating bootstrap-derived correlation stability coefficients of 0.25 (but preferably over 0.5) as the minimum for inferring stability of centrality metrics [16]. Stability of sample edge-weights derived from non-parametric bootstrapping was evaluated via visual comparison of sample and mean bootstrapped edge-weights. Community analysis in the resulting network was performed using the *igraph* [13] package by maximising the modularity measure over all possible partitions of the network using the “cluster_optimal” function [6]. Modularity is a measure of the extent of separation between nodes from different communities within a network, such that a modularity score of 0 represents that edges between nodes from different communities are just as numerous and strong as edges between nodes from the same community, whilst values approaching 1 reflect a strong community structure [38]. The bridge-strength centrality of each node, indicating the extent to which each node “bridged” the different communities of nodes, was calculated using the *networktools* package [22, 23]. These analytic steps were undertaken in the whole sample, and in boys and girls separately. The networks for boys and girls were statistically compared using the *NetworkComparisonTest* package [44]. Analyses stratified by age into early (0–5 years) and middle (6–12 years) childhood were considered, but not pursued due to the limited disorder cases in early childhood (Table S1). To ensure that comorbid conditional associations reflected the likelihood of comorbidity in the general population and to avoid Berkson’s bias [14], children with no recorded mental disorders were included in analyses, but were coded as 0 on all mental disorder indicator variables. Where the frequency of children with a particular disorder was n < 15, this number was suppressed to mitigate the risk of potential reidentification of children within the cohort. The syntax for these analyses can be found on the Open Science Network (https://osf.io/tkngm/?view_only=2acb980526d9432db6f78506209479ad).

## Results

The number of children with each type of mental disorder among the 90,269 children included in the network analysis, both overall and for boys (*n* = 46,732) and girls (*n* = 43,537) separately, is displayed in Table 1; 2268 children had at least one of the examined mental disorder diagnoses by 12 years of age. Of these 2268 children, 461 (20.3%) had two or more different types of disorders, 126 (5.6%) had more than two disorders, and 51 (2.2%) had more than three disorders. The distribution of age at the time of each presentation was negatively skewed for most disorders; that is, most service provision for mental disorders occurred later in childhood (see Figure S1). In contrast, most sleep disorder diagnoses occurred prior to 4 years of age. The majority of children included in the analysis were not diagnosed with any of the examined mental disorders (*n* = 88,001).

**Table 1:** Frequencies of mental disorder diagnoses among children evident in health records prior to age 12 years

Mental disorder category	Overall (*n* = 90,269)	Boys (*n* = 46,732)	Girls (*n* = 43,537)
	N (column %)	N (column %)	N (column %)
DDS	632 (0.70)	460 (0.98%)	172 (0.40%)
ANX	531 (0.58)	273 (0.58%)	258 (0.59%)
SLD	479 (0.53)	262 (0.56%)	217 (0.50%)
CAE	391 (0.43)	245 (0.52%)	146 (0.34%)
CON	330 (0.37)	241 (0.52%)	89 (0.20%)
HYP	227 (0.25)	174 (0.37%)	53 (0.12%)
STR	194 (0.21)	116 (0.25%)	78 (0.18%)
IND	136 (0.15)	87 (0.19%)	49 (0.11%)

*DDS* developmental disorders, *ANX* anxiety/phobia disorders, *SLD* sleep disorders, *CAE* childhood affective/emotional disorders, *CON* conduct disorders, *HYP* hyperkinetic disorders, *STR* stress reactions, *IND* intellectual disability

### Undirected weighted network of mental disorders in children

**Fig. 1:**
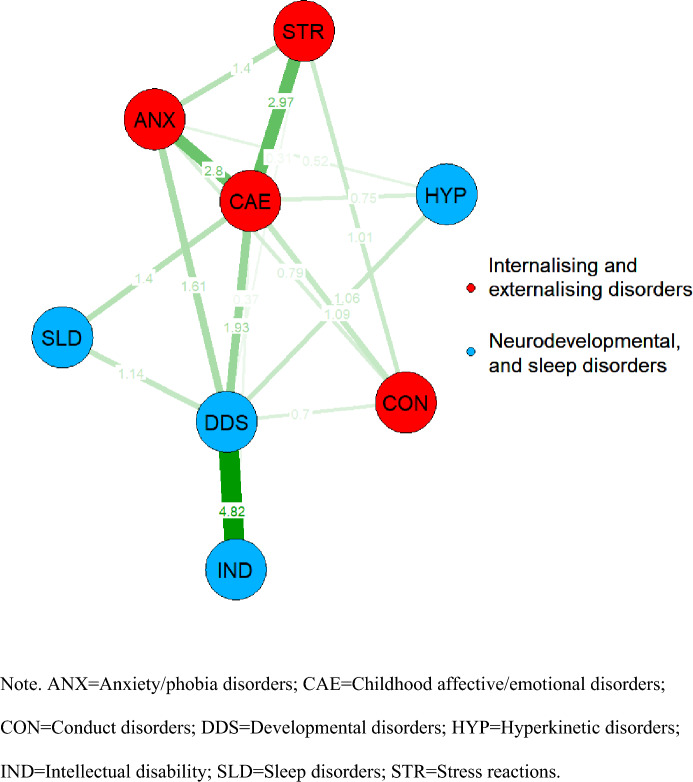
Undirected network of mental disorder comorbidity among children. *ANX* anxiety/phobia disorders, *CAE* childhood affective/emotional disorders, *CON* conduct disorders, *DDS* developmental disorders, *HYP* hyperkinetic disorders, *IND* intellectual disability, *SLD* sleep disorders, *STR* stress reactions

**Fig. 2:**
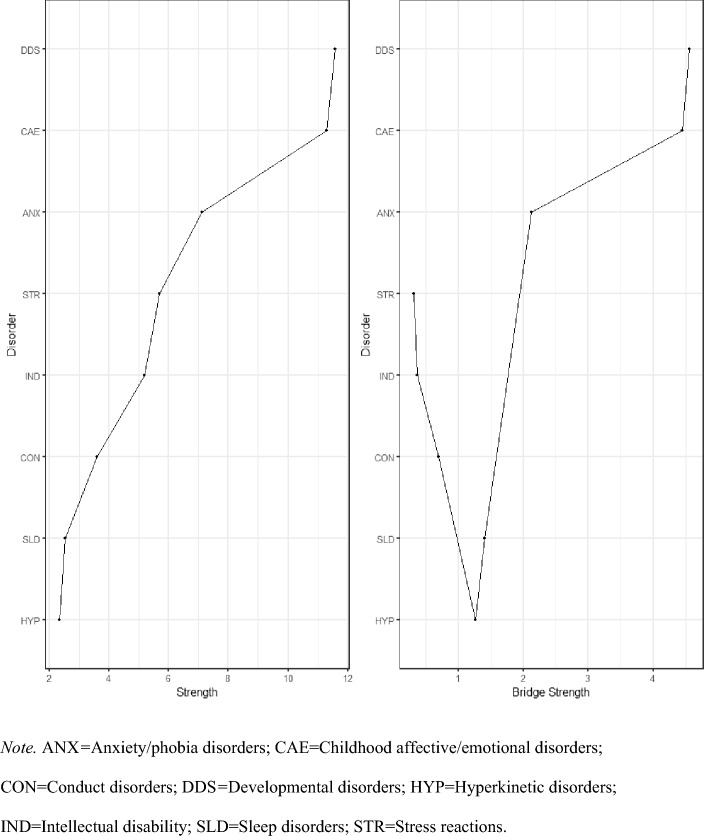
Strength centrality and bridge-strength centrality for mental disorder comorbidity network nodes. *ANX* anxiety/phobia disorders, *CAE* childhood affective/emotional disorders, *CON* conduct disorders, *DDS* developmental disorders, *HYP* hyperkinetic disorders, *IND* intellectual disability, *SLD* sleep disorders, *STR* stress reactions

The undirected weighted network of mental disorder comorbidity in children prior to 12 years of age is displayed in Fig. 1 (edge-weights also displayed in Table S3), with corresponding centrality and bridge strength reported in Fig. 2. The maximum modularity clustering algorithm converged on a solution which partitioned internalising (childhood affective and emotional disorders, stress reactions, and anxiety and phobic disorders) and externalising (conduct) mental disorder categories into one community, and neurodevelopmental (including developmental, hyperkinetic, and intellectual disorders) and sleep disorders into another community (modularity = 0.185). Developmental disorders and child affective and emotional disorders were the most central nodes in the network, followed by anxiety and phobic disorders, both in terms of their strength and bridge-strength centrality, reflecting that these disorder nodes were associated with other nodes within and across their respective disorder communities. In contrast, while stress reactions and intellectual disabilities were the next most central in terms of their strength centrality, they were the weakest nodes in terms of their bridge-strength centrality. Hyperkinetic, sleep, and conduct disorders were all relatively weak in terms of their centrality and bridge strength. Both edge-weights and centrality matrices were stable according to bootstrapped estimates (see Figures S2 and S3).

### Mental disorder networks separated by sex

**Fig. 3:**
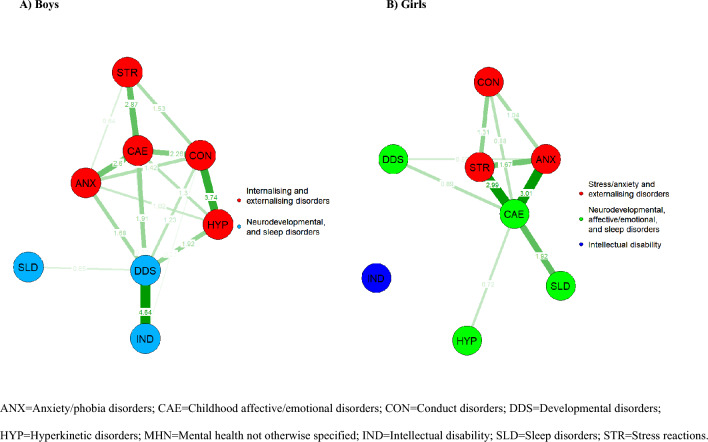
Mental disorder cumulative comorbidity network presented for A boys and B girls separately. *ANX* anxiety/phobia disorders, *CAE* childhood affective/emotional disorders, *CON* conduct disorders, *DDS* developmental disorders, *HYP* hyperkinetic disorders, *MHN* mental health not otherwise specified, *IND* intellectual disability, *SLD* sleep disorders, *STR* stress reactions

**Fig. 4:**
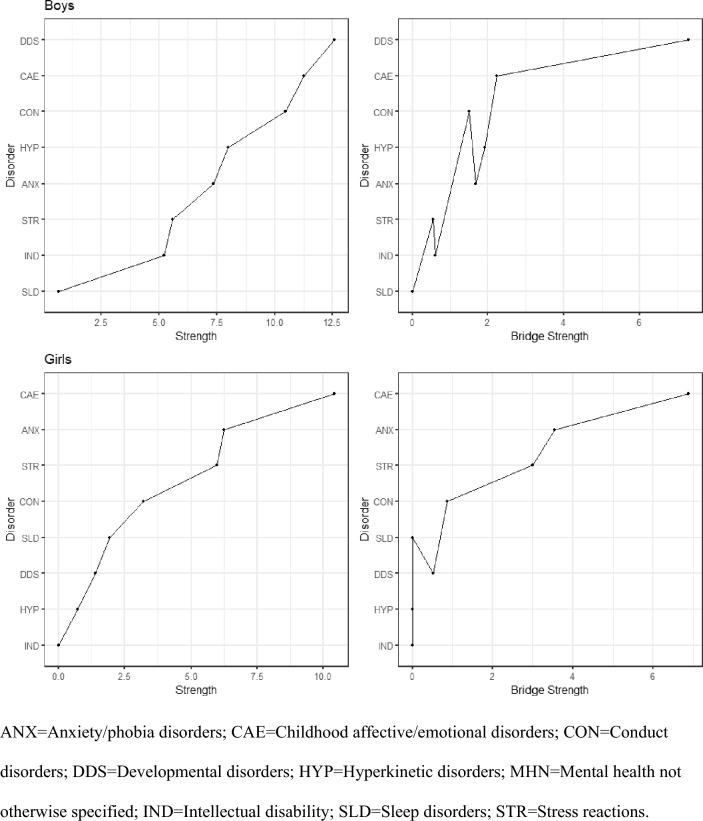
Strength and bridge-strength centrality metrics for mental disorder networks in boys and girls. *ANX* anxiety/phobia disorders, *CAE* childhood affective/emotional disorders, *CON* conduct disorders, *DDS* developmental disorders, *HYP* hyperkinetic disorders, *MHN* mental health not otherwise specified, *IND* intellectual disability, *SLD* sleep disorders, *STR* stress reactions

The mental disorder networks for boys and girls are displayed in Fig. 3 (edge-weights also displayed in Table S3) with corresponding centrality and bridge strength metrics for each node reported in Fig. 4. The network for boys was similar to the general network, except that hyperkinetic disorders clustered with the internalising and externalising disorders (modularity = 0.164). In contrast, the mental disorder network for girls clustered into three communities, such that child affective and emotional disorders, hyperkinetic disorders, sleep disorders, and developmental disorders were in one community; anxiety/phobic disorders, stress reactions, and conduct disorders in another; with intellectual disability in a community of its own (modularity = 0.004). A network invariance test revealed that these networks were significantly different (*t* = 4.637, *p* < 0.01), with the boys’ network having a significantly higher global strength (30.56) relative to the girls’ network (14.95) (*t* = 15.61, *p* = 0.03). Greater strength centrality for developmental disorders (*p* < 0.01), hyperkinetic disorders (*p* = 0.02), and intellectual disabilities (*p* < 0.01) was also observed in the boys’ mental disorder network, relative to the girls’ (see Table S4). This, in turn, was underpinned by stronger edge-weights in the boys’ relative to the girls’ mental disorder network between conduct disorders and child affective and emotional disorders (*p* = 0.02), developmental disorders (*p* = 0.05), hyperkinetic disorders (*p* = 0.04); and between developmental disorders and intellectual disability (*p* < 0.01), and hyperkinetic disorders (*p* = 0.05) (see Table S5). Differences in bridge strength between the boys’ and girls’ mental disorder networks could not be formally tested, as the required r package does not currently support comparison across networks with different community structures. However, the bridge strength in both networks largely mirrored the strength centrality in each respective network. The robustness of edge-weights and centrality measures for the boys’ and girls’ mental disorder networks are displayed in Figures S4–S7.

## Discussion

Network analysis of mental disorder categories in children revealed pervasive cumulative comorbidity up to age 12 years. Developmental disorders and childhood affective and emotional disorders demonstrated the greatest extent of comorbidity with other disorders and were the most central to the network. Stress reactions, anxiety and phobic disorders, and intellectual disability were also moderately central to the network, whilst hyperkinetic and sleep disorders were the least central. Interestingly, while neurodevelopmental disorders (here including developmental disorders, intellectual disability, and hyperkinetic disorders) and sleep disorders clustered into a single community of disorder nodes, internalising (affective and emotional, anxiety and phobic, and stress reactions) and externalising (conduct) disorders all clustered together into a second single community. However, the extent of the division into network communities was weak, reflecting considerable comorbidity between neurodevelopmental disorders and internalising and externalising disorders. Significant differences were observed between mental disorder networks in boys and girls, such that developmental disorders, hyperkinetic disorders, and intellectual disability displayed significantly stronger centrality in the boys’ relative to the girls’ mental disorder network.

Borsboom and colleagues predicted that networks of mental disorders would aggregate into internalising and externalising disorders, consistent with hierarchical models (e.g. HiTOP) of psychopathology [5]. A subsequent network analysis of internalising and externalising disorders in childhood proved consistent with this hypothesis, finding that internalising and externalising disorders (which included both hyperkinetic and conduct disorders), whilst broadly comorbid, clustered into separate communities of disorders [35]. The findings of the present study—which included an extended range of childhood mental disorders—conflict with these findings, in that externalising (conduct) disorders clustered into a single community with internalising disorders (i.e., comprising childhood affective and emotional disorders, anxiety and phobic disorders, and stress reactions), while the other community of disorder nodes comprised sleep and neurodevelopmental disorders (i.e., developmental disorders, intellectual disability, and hyperkinetic disorders). One possibility for these discrepant findings is that there was only one externalising disorder node (conduct disorders) in the current network (noting that the limited number of cases precluded the examination of different types of externalising disorders), resulting in it clustering with internalising disorders in the absence of other externalising disorders with which to cluster. Furthermore, while our finding that hyperkinetic disorders clustered with neurodevelopmental disorders is inconsistent with the previous network analysis of psychopathology in childhood [35], it is consistent with evidence that hyperkinetic disorders are more closely related to neurodevelopmental disorders than to externalising or internalising problems [15]. The lack of inclusion of developmental disorders in the previous network analysis may thus have led to the clustering of hyperkinetic disorders with the next most closely related disorders—conduct disorder and oppositional defiant disorder [35]. Interestingly, hyperkinetic disorders also clustered with internalising and conduct disorders in boys when analysed alone, suggesting that there may also be important sex differences in mental disorder comorbidity which may have influenced our findings in the overall network. Whether these differences are underpinned by genuine sex differences in prevalence or gender biases in diagnostic instruments remains an area of ongoing research and debate [28]. The previous network analysis also assessed children’s mental illness based on parental reports obtained via survey [35], in contrast to the current study which ascertained the mental health diagnoses of children from health records. While the previous network analyses may thus have been subject to selection and other biases related to the accuracy of parental report [2], the current study may have been biased in other ways; for example, the children with mental disorder diagnoses in the current study likely represent the severe end of the clinical spectrum given their reliance on hospital and mental-health ambulatory services for the treatment of mental disorders [30].

From a network perspective of psychopathology, the fact that developmental disorders and childhood affective and emotional disorders were the most central to the network could suggest that these mental disorder categories share the most “bridge” symptoms with the other psychiatric disorders examined [4]. Indeed, these two groups of mental disorder categories also had the highest bridge centrality, suggesting that these disorder categories were also more likely to be comorbid with disorder categories from the other disorder community. The strong comorbidity between childhood affective and emotional disorders, anxiety and phobic disorders, and stress reactions are unsurprising given that these disorder categories share many symptoms in common and that distinct symptoms of these disorder categories frequently coexist [42]. In contrast, while the developmental disorders node was most strongly connected to the intellectual disability node, it was also the only node which shared edges with every other disorder node in the network. High rates of comorbidity between developmental disorders (particularly pervasive developmental disorders) and intellectual disability [33], internalising and externalising problems [41], hyperkinetic disorders [27], and sleep disorders [12], have all been reported previously. Other network analyses of children with autism have consistently observed many of these same comorbidities [18, 32]. Evidence suggests that comorbidity between neurodevelopmental disorders and various forms of psychopathology may be underpinned by shared genetic and environmental risk factors [18, 37, 45]. There is further evidence, at least in the case of hyperkinetic and anxiety disorders, that comorbidity between pervasive developmental disorders and other forms of psychopathology may be underpinned by common, mutually reinforcing “bridge” symptoms [18, 46]. However, to our knowledge, no other network analysis has observed conditional associations between developmental disorders and externalising (conduct) disorders, despite such comorbidities being reported in the broader literature [24]. The centrality of developmental disorders to the overall network suggests that a temporal network analysis of developmental, internalising, and externalising problems at the symptom-level may prove instrumental in elucidating the symptoms which bridge developmental disorders with other forms of psychopathology. Such temporal analyses would also allow for the calculation of bridge “outstrength” and “instrength”, providing metrics reflecting the extent to which each disorder may precede or succeed other diagnoses, potentially elucidating the causal relationships between disorders [23].

Interestingly, numerous differences were observed between mental disorder networks in girls and boys, such that the community structure of disorder categories in boys (which was similar to the community structure in the entire cohort) was stronger, with developmental, hyperkinetic, and intellectual disorders all being more central to the network. In contrast, the community structure in girls was very weak, suggesting that disorder categories were just as likely to be comorbid across as within their respective disorder “communities”. Alongside evidence of neuroanatomical sex differences in developmental disorders, these results must be considered in light of recent findings that the diagnosis of neurodevelopmental disorders in girls may occur later in development and at a reduced rate overall, suggesting potential sex biases may exist in the instruments used to diagnose these disorders [7]. Future research is needed to determine if these sex differences in network structure persist into later adolescence and adulthood.

The extent of cumulative comorbidity between mental disorders in children may be largely an artefact of the prevailing categorical approach to psychiatric diagnosis, which naturally facilitates an accumulation of separate diagnoses over time. Alternatively, a more person-centred, developmental framework that took a dimensional approach to psychopathology might reveal a pattern of branching and proliferation of symptoms in relation to environmental influences over time. If we were to assume a hierarchy of psychopathology in which developmental disorders were regarded as primarily ‘internally’ (i.e., biologically) driven and the externalising/internalising disorders as primarily ‘externally’ (i.e., socially) driven, then the observations of bridging found in this study might be intelligible in terms of children with and without developmental disorders reacting to social circumstances (to which those with developmental disorders may be especially vulnerable) with emotional and behavioural symptoms that coalesce in the form of internalising and/or externalising diagnoses. Just as certain symptoms have been found to bridge different mental disorders [25, 31, 35], here child affective and emotional disorders and developmental disorders were found to bridge broader disorder communities of internalising and externalising, and neurodevelopmental disorders. Given that our findings represent cumulative comorbidity over the course of 12 years, the patterns of comorbidity described could be the result of the developmental dynamics of mental disorders shifting in their manifestations (symptoms branching and proliferating) over time and/or concurrent comorbid disorders emerging in relation to external events. Regardless, the presence of symptoms spanning multiple mental disorders should guide treatment that is well informed by the social conditions in which the symptoms of those disorders have been evoked. This may be a particularly important consideration for children with affective and emotional disorders, and developmental disorders, which seem to show pervasive patterns of comorbidity across the spectrum of psychopathology.

The present findings should be considered in light of several limitations. First, information on psychiatric diagnoses came from public health records (admitted patients, emergency department, and mental health ambulatory). As such, people who sought treatment for mental disorders outside of these three services (e.g., from general practitioners, psychologists, psychiatrists, or counsellors), in addition to those who never sought treatment, may have incorrectly been regarded as “healthy”. These data likely underestimate the true extent of mental disorders and mental disorder comorbidity in childhood. Furthermore, given the transitory nature of contact with health services, there was no way to ascertain whether each diagnosis represented a temporary or enduring disorder active at the time of the study. The use of binary indicators representing different types of mental disorders also precluded more nuanced structural analysis of how symptoms might be related. The structure of any network would also be influenced by the manner in which mental disorders included within the network were categorised. The disorder categories included within the network in the current study were selected to balance disorder specificity and statistical power, by analysing groups of disorders aggregated based on symptom similarity and previously reported comorbidity rates, to produce relatively homogenous disorder groups of sufficient sample size to ensure robust network estimation. However, analysis of more distinct disorder categories in a cohort with a higher prevalence of mental illness may help to further clarify the patterns of comorbidity in childhood. Nevertheless, it should be noted that while there are guidelines about the overall sample size necessary to estimate a network [11], there are no recommendations (to our knowledge) regarding the number of (positive or negative) cases needed for dichotomous variable nodes, to ensure accurate estimation of edge-weights and centrality metrics. Additionally, this study did not distinguish between concurrent and cumulative comorbidity as two of the three sources of information on children’s mental health only noted the primary diagnosis for a given presentation without detailing comorbid conditions. Finally, this study also did not investigate the temporal order in which cumulative comorbidity occurred, as the order in which disorders were recorded in hospital/ambulatory records would not necessarily have corresponded to the chronological order in which disorders were first diagnosed. Future research which comprehensively assesses mental health across time (e.g. via self- or parent-report) would be better placed to reliably investigate the temporal order of mental disorder diagnoses. These limitations should be counter-balanced against the relative strengths of the study afforded by record linkage methodology, including reduced selection and information biases, and the large sample size.

In conclusion, patterns of cumulative mental disorder comorbidity appear to be pervasive in children as young as 12 years old, with developmental and child affective and emotional disorders being most central to the network of mental disorder comorbidity. This reinforces the case for person-centred care [39] which can accommodate the presence of multiple comorbid conditions. Network approaches may prove instrumental in this goal, by identifying the symptom structure underpinning psychiatric comorbidities, thus facilitating the targeted delivery of interventions focused on addressing “bridge” and central disorder symptoms [3].

### Supplementary Information

Below is the link to the electronic supplementary material.

Supplementary file1 (DOCX 1181 KB)

## Data Availability

Privacy legislation and ethical restrictions placed on the use of deidentified multiagency linked government data limit access to the study data which cannot be made publicly available. Collaborative research may be possible dependent on scope and resources.
