# Supplementary material for: Cumulative comorbidity between neurodevelopmental, internalising, and externalising disorders in childhood: a network approach
Source: Eur Child Adolesc Psychiatry. 2023 Oct 10;33(7):2231–41. doi: 10.1007/s00787-023-02312-7 (PMC11255061; doi:10.1007/s00787-023-02312-7)

**Comorbidity between neurodevelopmental, internalising, and externalising disorders in childhood: A network approach**

Oliver J Watkeys, PhD^1,2^, Kirstie O’Hare, PhD^1^, Kimberlie Dean, PhD^1,3^, Kristin R Laurens PhD^1,4^, Felicity Harris, MAClinEpi ^1^, Vaughan J Carr, MD^1,2,5^, Melissa J Green, PhD^1,2^

**Author Affiliations**

1. School of Clinical Medicine, Discipline of Psychiatry and Mental Health, University of New South Wales (UNSW), Sydney, Australia

2. Neuroscience Research Australia, Sydney, Australia

3. Justice Health & Forensic Mental Network, Matraville, Australia

4. Queensland University of Technology (QUT), School of Psychology and Counselling, Brisbane, Australia

5. Department of Psychiatry, Monash University, Melbourne, Australia

**Table of Contents**

[Table S1. Frequencies for psychiatric disorder groups analysed, and the individual ICD-10-AM codes of which they were comprised. 2](#_Toc146193920)

[Table S2. Frequency of different mental disorders in early (0-5 years) and middle (6-12 years) childhood 3](#_Toc146193921)

[Table S3. Network edge-weights between mental disorders in children up to age 12 years 3](#_Toc146193922)

[Table S4. Strength-centrality invariance test between networks in boys and girls 4](#_Toc146193923)

[Table S5. Edge-weight invariance test between mental disorder networks in boys and girls 4](#_Toc146193924)

[Figure S1. Age at time of any presentation for each type of mental disorder. 5](#_Toc146193925)

[Figure S2. Stability of network edge-weights derived via bootstrapping 6](#_Toc146193926)

[Figure S3. Stability of network centrality derived by bootstrapping across using different percentages of original sample. 7](#_Toc146193927)

[Figure S4. Stability of network edge-weights derived via bootstrapping for boy’s mental disorder network. 8](#_Toc146193928)

[Figure S5. Stability of network centrality derived by bootstrapping across using different percentages of original sample for boy’s mental disorder network. 9](#_Toc146193929)

[Figure S6. Stability of network edge-weights derived via bootstrapping for girl’s mental disorder network. 10](#_Toc146193930)

[Figure S7. Stability of network centrality derived by bootstrapping across using different percentages of original sample for girl’s mental disorder network. 11](#_Toc146193931)

| Table S1. Frequencies for psychiatric disorder groups analysed, and the individual ICD-10-AM codes of which they were comprised. | | | |
| --- | --- | --- | --- |
| **Name** | **Acronym** | **Code** | **N** |
| Developmental disorders | DDS | Total | 632 |
|  |  | F80 (Specific developmental disorders of speech and language) | 156 |
|  |  | F81 (Specific developmental disorders of scholastic skills) | 56 |
|  |  | F82 (Specific developmental disorder of motor function) | 21 |
|  |  | F83 (Mixed specific developmental disorders) | 72 |
|  |  | F84 (Pervasive developmental disorders) | 346 |
|  |  | F88 (Other disorders of psychological development) | n<15 |
|  |  | F89 (Unspecified disorder of psychological development) | 108 |
| Anxiety/phobia disorders | ANX | Total | 531 |
|  |  | F40 (Phobic anxiety disorders) | 36 |
|  |  | F41 (Other anxiety disorders) | 504 |
| Sleep disorders | SLD | F51 (Nonorganic sleep disorders) | 479 |
| Childhood affective/emotional disorders | CAE | Total | 391 |
|  |  | F32 (Depressive episode) | 68 |
|  |  | F34 (Persistent mood disorders) | n<15 |
|  |  | F93 (Emotional disorders with onset specific to childhood) | 203 |
|  |  | F98 (Other behavioural and emotional disorders with onset usually occurring in childhood and adolescence) | 179 |
| Conduct disorders | CON | F91 (Conduct disorders) | 330 |
| Hyperkinetic disorders | HYP | F90 (Hyperkinetic disorders) | 227 |
| Stress reactions | STR | F43 (Reaction to severe stress, and adjustment disorders) | 194 |
| Intellectual disability | IND | Total | 136 |
|  |  | F70 (Mild mental retardation) | 17 |
|  |  | F71 (Moderate mental retardation) | n<15 |
|  |  | F72 (Severe mental retardation) | n<15 |
|  |  | F73 (Profound mental retardation) | n<15 |
|  |  | F78 (Other mental retardation) | n<15 |
|  |  | F79 (Unspecified mental retardation) | 113 |

Note: n<15 = minimum cell size conditions apply to the use of this data. Also note that disorders are not mutually exclusive, even within the broader disorder groups used in analyses. ICD-10-AM = International Classification of Diseases 10^th^ Edition Australian Modification.

# Table S2. Frequency of different mental disorders in early (0-5 years) and middle (6-12 years) childhood

|  | **0-5 years** | **6-12 years** |
| --- | --- | --- |
| **Disorder** | **n (column %)** | **n (column %)** |
| **Anxiety and phobia disorders (ANX)** | 32 (0.04%) | 508 (0.56%) |
| **Childhood affective and emotional disorders (CAE)** | 55 (0.06%) | 355 (0.39%) |
| **Conduct disorders (CON)** | 76 (0.08%) | 264 (0.29%) |
| **Developmental disorders (DDS)** | 279 (0.31%) | 447 (0.50%) |
| **Hyperkinetic disorders (HYP)** | 23 (0.03%) | 211 (0.23%) |
| **Intellectual disability (IND)** | 69 (0.08%) | 82 (0.09%) |
| **Sleep disorders (SLD)** | 460 (0.51%) | 23 (0.03%) |
| **Stress reactions (STR)** | 22 (0.02%) | 180 (0.20%) |

# Table S3. Network edge-weights between mental disorders in children up to age 12 years

|  | **ANX** | **CAE** | **CON** | **DDS** | **HYP** | **IND** | **SLD** |
| --- | --- | --- | --- | --- | --- | --- | --- |
|  | **Overall** | | | | | | |
| **CAE** | 2.80 |  |  |  |  |  |  |
| **CON** | 0.79 | 1.09 |  |  |  |  |  |
| **DDS** | 1.61 | 1.93 | 0.70 |  |  |  |  |
| **HYP** | 0.52 | 0.75 | 0.00 | 1.06 |  |  |  |
| **IND** | 0.00 | 0.37 | 0.00 | 4.82 | 0.00 |  |  |
| **SLD** | 0.00 | 1.40 | 0.00 | 1.14 | 0.00 | 0.00 |  |
| **STR** | 1.40 | 2.97 | 1.01 | 0.31 | 0.00 | 0.00 | 0.00 |
|  | **Boys** | | | | | | |
| **CAE** | 2.60 |  |  |  |  |  |  |
| **CON** | 1.42 | 2.26 |  |  |  |  |  |
| **DDS** | 1.68 | 1.91 | 1.23 |  |  |  |  |
| **HYP** | 1.02 | 1.30 | 3.74 | 1.92 |  |  |  |
| **IND** | 0.00 | 0.32 | 0.27 | 4.64 | 0.00 |  |  |
| **SLD** | 0.00 | 0.00 | 0.00 | 0.65 | 0.00 | 0.00 |  |
| **STR** | 0.64 | 2.87 | 1.53 | 0.55 | 0.00 | 0.00 | 0.00 |
|  | **Girls** | | | | | | |
| **CAE** | 3.01 |  |  |  |  |  |  |
| **CON** | 1.04 | 0.88 |  |  |  |  |  |
| **DDS** | 0.52 | 0.89 | 0.00 |  |  |  |  |
| **HYP** | 0.00 | 0.72 | 0.00 | 0.00 |  |  |  |
| **IND** | 0.00 | 0.00 | 0.00 | 0.00 | 0.00 |  |  |
| **SLD** | 0.00 | 1.92 | 0.00 | 0.00 | 0.00 | 0.00 |  |
| **STR** | 1.67 | 2.99 | 1.31 | 0.00 | 0.00 | 0.00 | 0.00 |

*Note.* ANX *=* Anxiety/phobia disorders; CAE = Childhood affective/emotional disorders; CON = Conduct disorders; DDS = Developmental disorders; HYP = Hyperkinetic disorders; IND = Intellectual disability; SLD = Sleep disorders; STR = Stress reactions.

# Table S4. Strength-centrality invariance test between networks in boys and girls

| **Disorder** | **Strength centrality in boys** | **Strength centrality in girls** | **p-value for strength-centrality invariance test** |
| --- | --- | --- | --- |
| ANX | 7.35 | 6.24 | 0.40 |
| CAE | 11.26 | 10.42 | 0.66 |
| CON | 10.45 | 3.23 | 0.1 |
| DDS | 12.58 | 1.41 | <0.01 |
| HYP | 7.99 | 0.72 | 0.02 |
| IND | 5.24 | 0.00 | <0.01 |
| SLD | 0.65 | 1.92 | 0.4 |
| STR | 5.59 | 5.97 | 0.81 |

*Note:* ANX = Anxiety/phobia disorders; CAE = Childhood affective/emotional disorders; CON = Conduct disorders; DDS = Developmental disorders; HYP = Hyperkinetic disorders; MHN = Mental health not otherwise specified; IND = Intellectual disability; SLD = Sleep disorders; STR = Stress reactions.

# Table S5. Edge-weight invariance test between mental disorder networks in boys and girls

| **From** | **To** | **Edge-weight in boys** | **Edge-weight in girls** | **p-value** |
| --- | --- | --- | --- | --- |
| ANX | CAE | 2.60 | 3.01 | 0.28 |
| ANX | CON | 1.42 | 1.04 | 0.26 |
| CAE | CON | 2.26 | 0.88 | 0.02 |
| ANX | DDS | 1.68 | 0.52 | 0.12 |
| CAE | DDS | 1.91 | 0.89 | 0.39 |
| CON | DDS | 1.23 | 0.00 | 0.05 |
| ANX | HYP | 1.02 | 0.00 | 0.13 |
| CAE | HYP | 1.30 | 0.72 | 0.28 |
| CON | HYP | 3.74 | 0.00 | 0.04 |
| DDS | HYP | 1.92 | 0.00 | 0.05 |
| ANX | IND | 0.00 | 0.00 | 1.00 |
| CAE | IND | 0.32 | 0.00 | 0.68 |
| CON | IND | 0.27 | 0.00 | 0.34 |
| DDS | IND | 4.64 | 0.00 | 0.00 |
| HYP | IND | 0.00 | 0.00 | 1.00 |
| ANX | SLD | 0.00 | 0.00 | 1.00 |
| CAE | SLD | 0.00 | 1.92 | 0.09 |
| CON | SLD | 0.00 | 0.00 | 1.00 |
| DDS | SLD | 0.65 | 0.00 | 0.53 |
| HYP | SLD | 0.00 | 0.00 | 1.00 |
| IND | SLD | 0.00 | 0.00 | 1.00 |
| ANX | STR | 0.64 | 1.67 | 0.39 |
| CAE | STR | 2.87 | 2.99 | 0.91 |
| CON | STR | 1.53 | 1.31 | 0.73 |
| DDS | STR | 0.55 | 0.00 | 0.19 |
| HYP | STR | 0.00 | 0.00 | 1.00 |
| IND | STR | 0.00 | 0.00 | 1.00 |
| SLD | STR | 0.00 | 0.00 | 1.00 |

*Note:* ANX = Anxiety/phobia disorders; CAE = Childhood affective/emotional disorders; CON = Conduct disorders; DDS = Developmental disorders; HYP = Hyperkinetic disorders; MHN = Mental health not otherwise specified; IND = Intellectual disability; SLD = Sleep disorders; STR = Stress reactions.

Figure S1. Age at time of any presentation for each type of mental disorder. *Note*. ANX = Anxiety/phobia disorders; CAE = Childhood affective/emotional disorders; CON = Conduct disorders; DDS = Developmental disorders; HYP = Hyperkinetic disorders; MHN = Mental health not otherwise specified; IND = Intellectual disability; SLD = Sleep disorders; STR = Stress reactions.


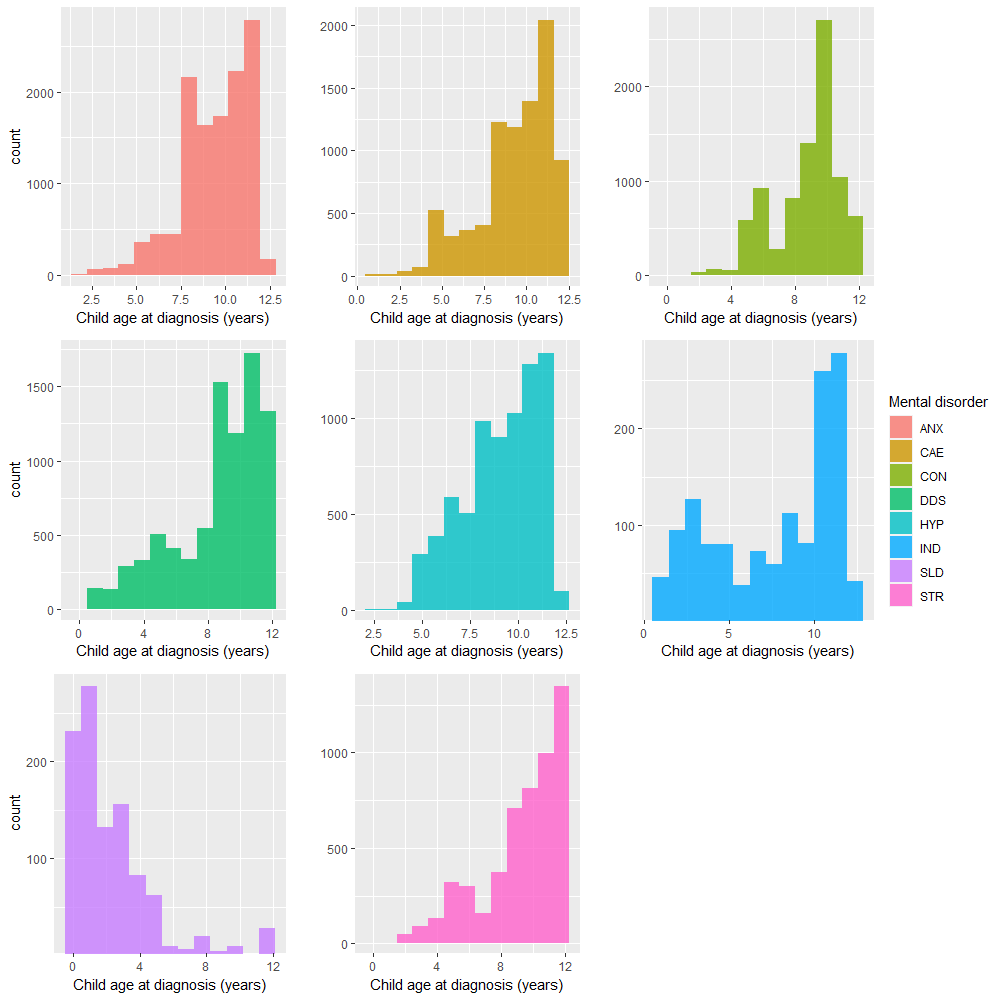


Figure S2. Stability of network edge-weights derived via bootstrapping
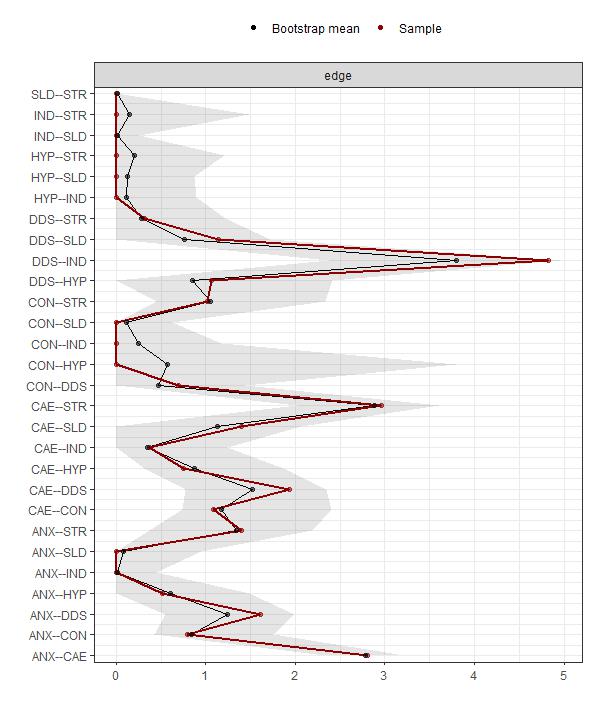


*Note:* ANX = Anxiety/phobia disorders; CAE = Childhood affective/emotional disorders; CON = Conduct disorders; DDS = Developmental disorders; HYP = Hyperkinetic disorders; MHN = Mental health not otherwise specified; IND = Intellectual disability; SLD = Sleep disorders; STR = Stress reactions.

# Figure S3. Stability of network centrality derived by bootstrapping across using different percentages of original sample.


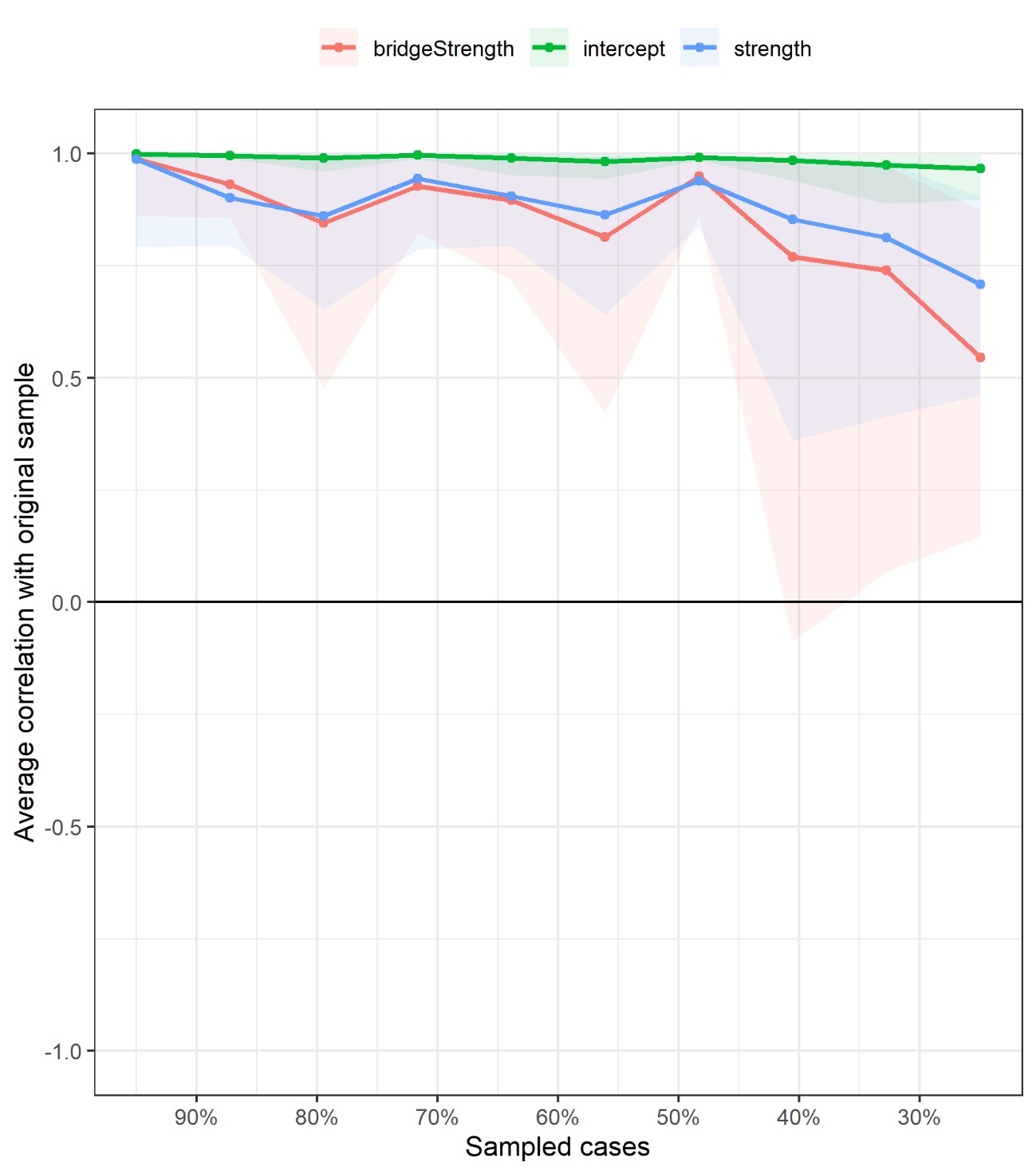


# Figure S4. **Stability of network edge-weights derived via bootstrapping for boy’s mental disorder network.**


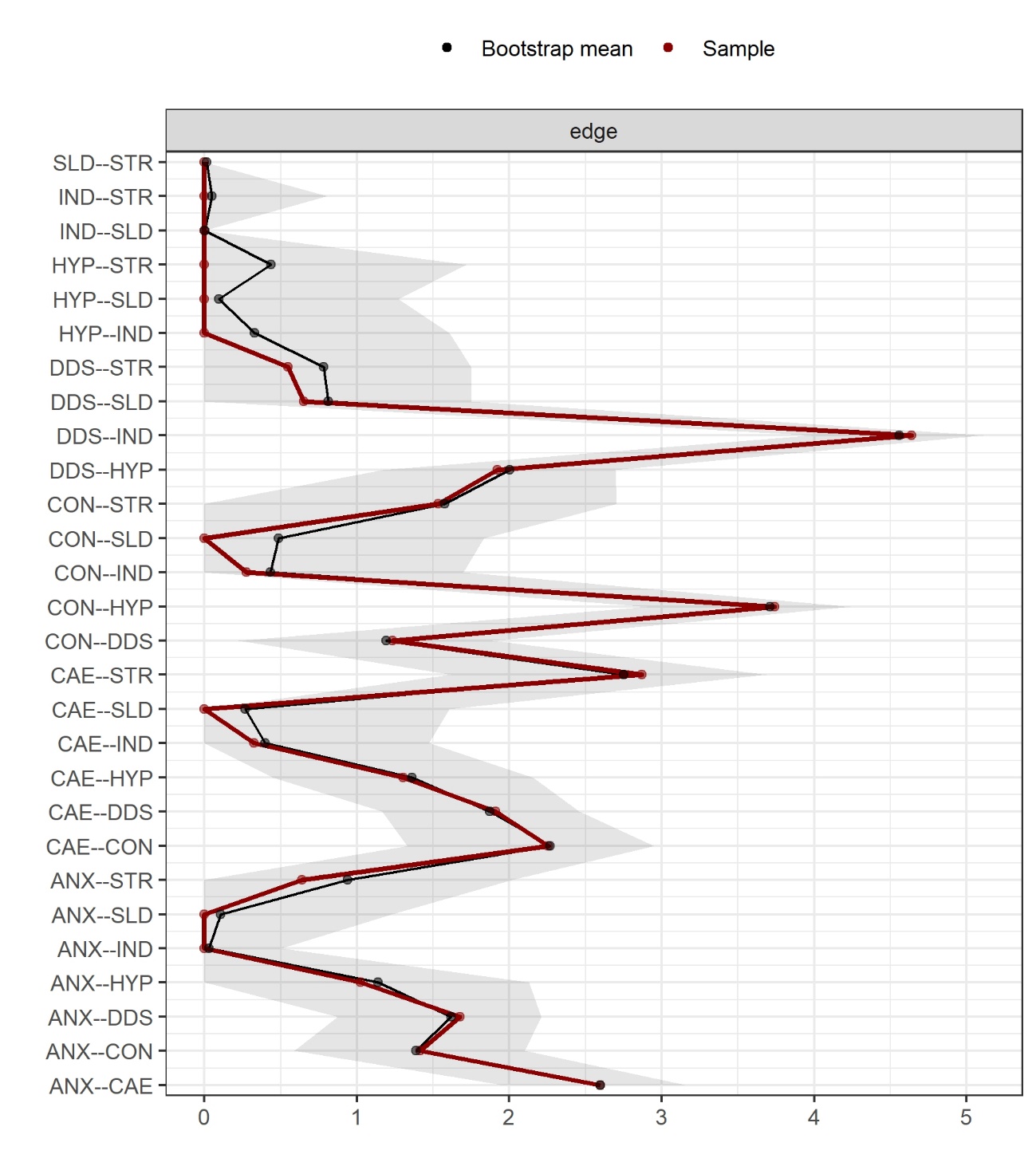


*Note:* ANX = Anxiety/phobia disorders; CAE = Childhood affective/emotional disorders; CON = Conduct disorders; DDS = Developmental disorders; HYP = Hyperkinetic disorders; MHN = Mental health not otherwise specified; IND = Intellectual disability; SLD = Sleep disorders; STR = Stress reactions.

# Figure S5. Stability of network centrality derived by bootstrapping across using different percentages of original sample for boy’s mental disorder network.


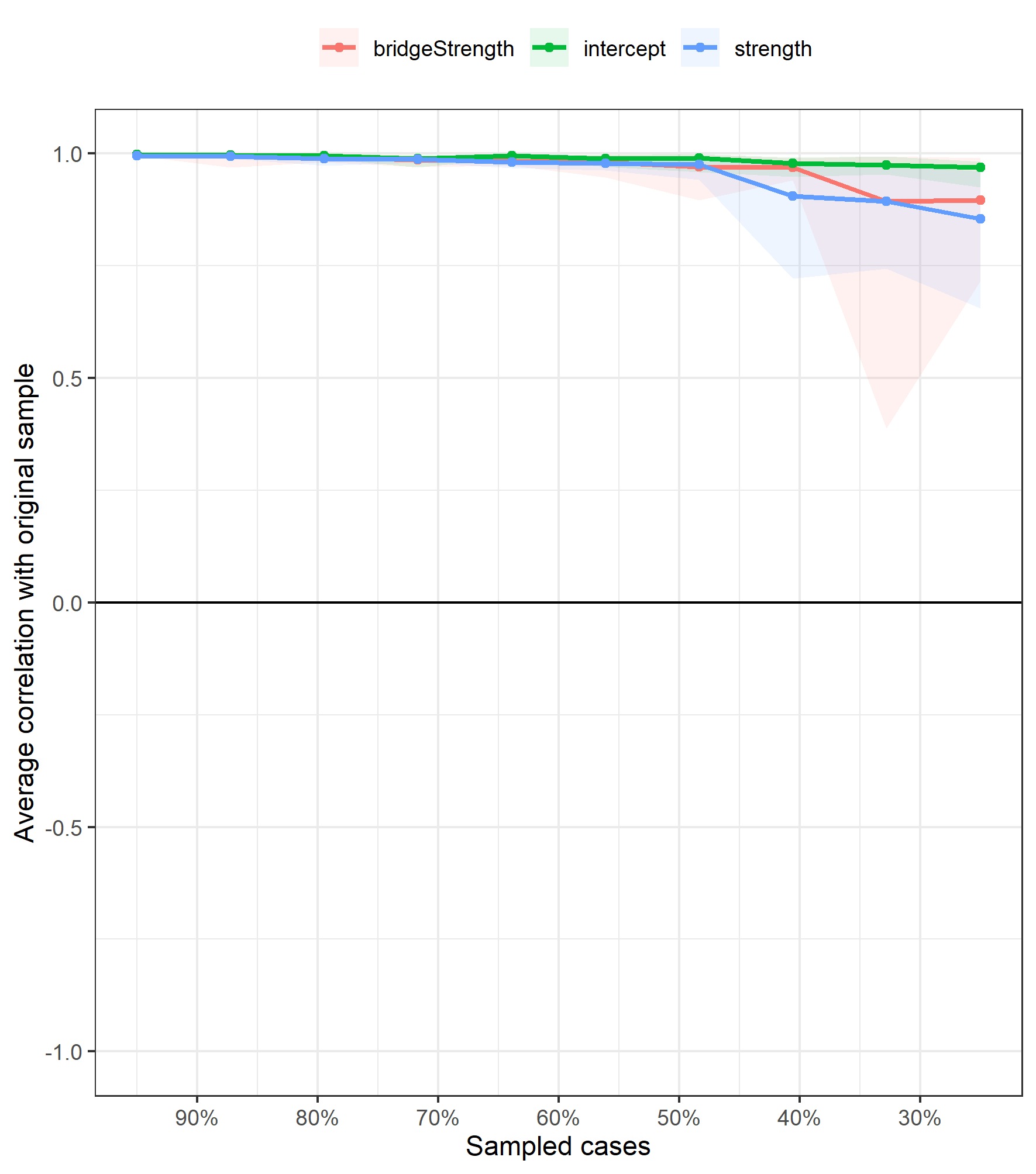


# Figure S6. **Stability of network edge-weights derived via bootstrapping for girl’s mental disorder network.**


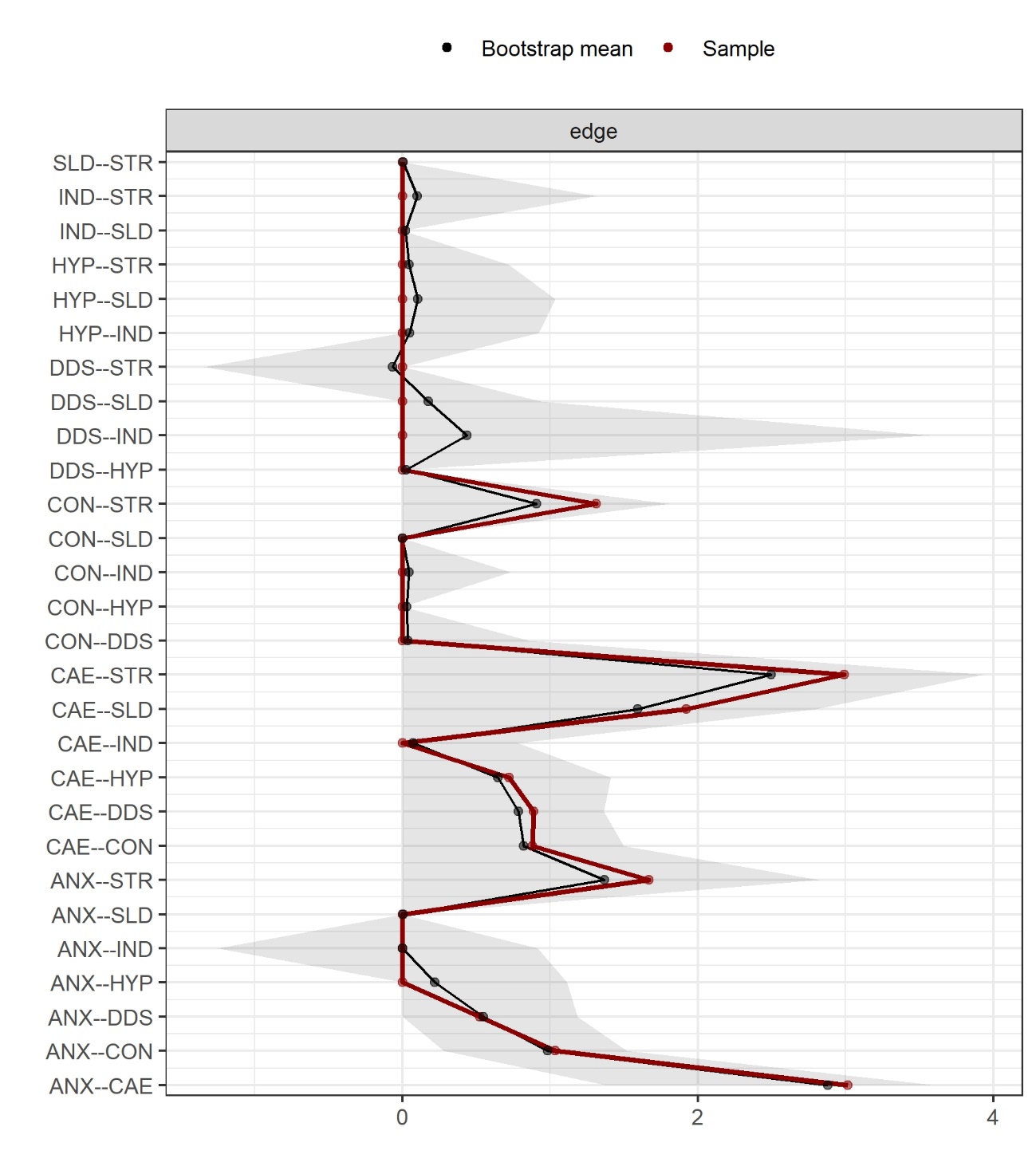


*Note:* ANX = Anxiety/phobia disorders; CAE = Childhood affective/emotional disorders; CON = Conduct disorders; DDS = Developmental disorders; HYP = Hyperkinetic disorders; MHN = Mental health not otherwise specified; IND = Intellectual disability; SLD = Sleep disorders; STR = Stress reactions.

# Figure S7. Stability of network centrality derived by bootstrapping across using different percentages of original sample for girl’s mental disorder network.


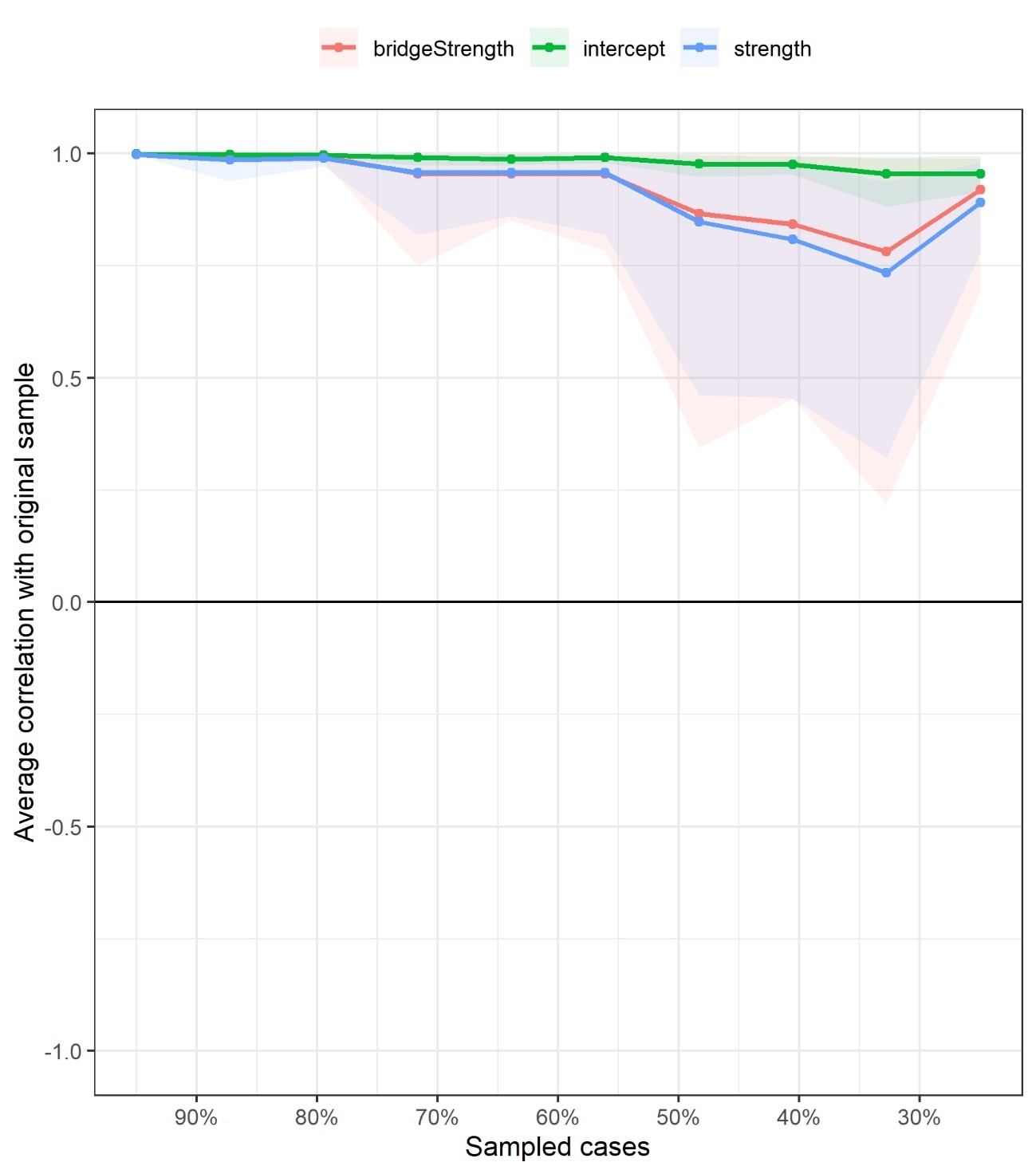

Supplement: Supplementary file 1 — Supplementary file1 (DOCX 1181 KB) [file 787_2023_2312_MOESM1_ESM.docx]
